# Chemical Markers and Bioactivity of Algerian Royal Jelly: Influence of Bee Subspecies, Geography, and Lyophilization

**DOI:** 10.1002/cbdv.71406

**Published:** 2026-06-11

**Authors:** A. S. Ayad, M. P. A. Hébert, J. A. Doiron, W. Loucif‐Ayad, T. Daas, G. Smagghe, M. Touaibia, M. E. Surette

**Affiliations:** ^1^ Pharmaceutical Sciences Research Center (CRSP) Constantine Algeria; ^2^ New Brunswick Centre For Precision Medicine Moncton New Brunswick Canada; ^3^ Department of Chemistry and Biochemistry Université De Moncton Moncton New Brunswick Canada; ^4^ New Brunswick Institute of Health Research Moncton New Brunswick Canada; ^5^ Laboratory of Applied Animal Biology, Faculty of Sciences Badji Mokhtar University Annaba Algeria; ^6^ Faculty of Medicine Badji Mokhtar University Annaba Algeria; ^7^ Institute of Entomology Guizhou University Guiyang China; ^8^ Department of Biology Vrije Universiteit Brussel (VUB) Brussels Belgium

**Keywords:** 10‐Hydroxy‐2‐decenoic acid (10‐HDA), Algerian honeybees, antioxidant activity, biodiversity, lyophilization, phenolic compounds, royal jelly

## Abstract

Royal jelly (RJ) is a chemically complex bee‐derived secretion whose composition and biological properties are influenced by honeybee genetics, geography, and processing. Native Algerian honeybee subspecies of the African lineage (*Apis mellifera intermissa* and *Apis mellifera sahariensis*) represent an underexplored source of RJ with potential chemical distinctiveness. In this study, fresh and lyophilized RJ samples from three geographically contrasting Algerian regions were characterized using selected chemical markers to assess biodiversity‐ and processing‐related effects. Chemical analyses revealed marked regional variability in 10‑hydroxy‑2‑decenoic acid, with fresh samples containing 1.9%–3.0% (w/w) and lyophilized samples reaching 4.9%–8.4% (w/w). Estimated total phenolic and flavonoid contents also varied by region. Fresh and lyophilized samples showed comparable patterns for the measured markers, supporting the use of both forms for comparative studies. Biological evaluation revealed weak antiradical and antioxidant activities. In cell‐based assays, lyophilized RJ selectively reduced Reh cell viability, while Jurkat cells were largely unaffected, with effects attributable to nonspecific cytotoxicity. RJ showed little to no inhibition of leukotriene biosynthesis in a whole‐cell 5‑lipoxygenase assay, indicating limited anti‐inflammatory activity via this pathway. By integrating selected chemical characterization with biologically relevant cellular assays, this work provides a balanced and mechanistically informed assessment of RJ bioactivity.

## Graphical abstract

Algerian royal jelly from native honey bees shows chemical diversity influenced by geography and subspecies. Lyophilization preserves key compounds, including 10‐HDA and phenolics. Biological assays reveal modest antioxidant activity and selective cytotoxicity against Reh leukemia cells, while anti‐inflammatory effects are limited. This study links biodiversity, processing, and bioactivity, highlighting lyophilized RJ as a stable natural product with region‐specific chemical signatures.

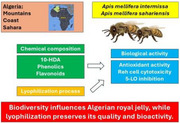

## 1 Introduction

Honeybees (*Apis mellifera*) produce a diverse array of natural products that occupy a unique position at the interface of chemistry and biology and are widely exploited in the pharmaceutical, food, and cosmetic industries due to their nutritional and health‐promoting properties [1]. The chemical composition and biological activities of these products are strongly influenced by ecological, genetic, and processing factors, making them compelling subjects for biodiversity‐driven chemical research [2, 3, 4]. In Algeria, two endemic honeybee subspecies belonging to the African lineage (A), *Apis mellifera intermissa* and *Apis mellifera sahariensis*, have been identified [5, 6]. The conservation of these native subspecies has motivated the establishment of queen breeding stations, which simultaneously support biodiversity preservation and the controlled production of high‐value bee products such as royal jelly (RJ).

RJ is a yellowish‐white, acidic secretion with a gelatinous consistency, produced by the hypopharyngeal and mandibular glands of nurse bees aged 3–12 days [7, 8]. It is the exclusive diet of queen bees throughout their lifespan and is fed transiently to worker larvae, resulting in profound phenotypic divergence between queens and workers, including differences in body size, fertility, and longevity [9]. From a chemical perspective, RJ represents a complex natural matrix composed primarily of water (50%–70%), proteins (9%–18%), carbohydrates (7%–18%), lipids (3%–8%), minerals, vitamins, and a variety of low‐molecular‐weight bioactive compounds [10]. This molecular complexity underlies the broad range of biological activities attributed to RJ in the literature, particularly antioxidant [11], antitumor [12] and anti‐inflammatory [13], which are the most relevant to the endpoints investigated in the present study. Although additional activities have been reported in different studies, such as antiviral [14], anti‐aging [15], neuroprotective [16], antimicrobial [17], hypocholesterolemic [18], and neurobehavioral effects [19], they were not addressed in the present study.

A defining chemical feature of RJ is the presence of 10‐hydroxy‐2‐decenoic acid (10‐HDA), a medium‐chain fatty acid unique to RJ and absent from other bee products and natural sources [20, 21]. Accounting for more than half of RJ's free fatty acid fraction, 10‐HDA has been proposed as both a biomolecular marker of RJ authenticity and a key contributor to its biological activities. Importantly, the abundance of 10‐HDA varies with geographical origin, environmental conditions, and honeybee subspecies, positioning RJ as a valuable system for exploring how biodiversity shapes chemical composition and biological function. In addition to 10‐HDA, major RJ proteins (MRJPs) [22], phenolic compounds, and micronutrients also contribute to RJ bioactivity, likely through additive or synergistic mechanisms [9, 23].

Beyond fresh RJ, lyophilized RJ is increasingly used in commercial formulations due to its improved stability, extended shelf life, and ease of storage at ambient temperatures, as lyophilization has been shown to preserve key RJ characteristics [24]. However, its inclusion in comparative studies remains useful to determine whether the principal chemical and biological patterns are conserved across sample forms derived from distinct ecological and genetic backgrounds. This is particularly relevant in the context of functional foods and nutraceuticals, where biological efficacy is often inferred from composition rather than being rigorously tested.

Motivated by these considerations, the present study was designed to address several interrelated biological questions: (i) how does geographical origin and associated honeybee subspecies influence the selected chemical profile of Algerian RJ? and (ii) are these chemical differences reflected in measurable differences in biological activity? For a stronger comparison, fresh and lyophilized RJ samples were analyzed in parallel to verify whether the main compositional and bioactivity patterns were maintained across the lyophilization process. Rather than measuring a comprehensive chemical profile of RJ, the present study focused on selected chemical markers relevant to RJ quality and bioactivity, namely 10‐HDA, total phenolic content, and total flavonoid content. To test these hypotheses, fresh and lyophilized RJ samples from three Algerian queen breeding stations were chemically characterized for 10‐HDA, total phenolic, and flavonoid contents. Their antiradical and antioxidant capacities were assessed, and selected cellular bioassays were employed to evaluate antiproliferative, pro‐apoptotic, and anti‐inflammatory activities using human leukemia cell lines and a cellular 5‐lipoxygenase (5‐LO) product biosynthesis model. By integrating selected chemical marker analysis with biologically relevant assays, the present work provides new insights into how biodiversity and geographical context shape the measured chemical traits and biological properties of RJ, with fresh and lyophilized samples serving as complementary forms within the experimental design, thereby contributing to a more nuanced understanding of RJ as a chemically complex natural product rather than a uniform bioactive substance.

## 2 Materials and Methods

### 2.1 Chemicals

All solvents, ethanol, methanol, and dimethyl sulfoxide (DMSO), were purchased from VWR Canada (Mississauga, ON, Canada). Folin‐Ciocalteu reagent, 2,2‐bipyridyl, 2,2‐diphenyl‐1‐picrylhydrazyl radical (DPPH•), quercetin, gallic acid, and calcium ionophore A23187 were obtained from Sigma–Aldrich (Oakville, ON, Canada). Arachidonic acid was purchased from Cayman Chemical (Ann Arbor, MI, USA). The 10‐HDA analytical standard was obtained from TargetMol Chemicals (Wellesley Hills, MA, USA). The CellTiter‐Blue reagent used for cell viability assays was purchased from Promega (Madison, WI, USA).

### 2.2 RJ Sample Collection and Preparation

Fresh RJ samples were collected from three queen breeding stations located in Annaba (GFA), situated in the Mediterranean coastal region; Médéa (GFM), located in the northern transitional hill zone; and Ghardaïa (GFG), located in the arid southern desert region of Algeria. The honeybee subspecies present in the Annaba and Médéa apiaries was *A. m. intermissa*, whereas *A. m. sahariensis* was identified in the Ghardaïa apiary. Sampling was conducted between March and April 2022 with the help of local beekeepers. For each geographical site, RJ from 5 to 6 hives was collected and pooled. The samples were immediately stored in the dark at −4°C, transported on dry ice, and handled under cold‐chain conditions to preserve sample integrity.

In parallel, the genetic status of honeybee populations from the three breeding stations was confirmed as belonging to the African lineage (A) using an in silico DraI mtDNA COI‐COII test, as previously described [25]. Lyophilized RJ samples from Annaba (GLA), Médéa (GLM), and Ghardaïa (GLG) were prepared by freeze‐drying the corresponding fresh samples using a Freeze Mobile 12ES Series freeze dryer. Samples were frozen at −20°C for 12 h and then subjected to lyophilization under a vacuum of 0.1 mbar for 48 h at a condenser temperature of −50°C.

Methanol was used as the solvent for chemical analyses, while DMSO was used for cellular assays. Fresh and lyophilized RJ samples were suspended in the appropriate solvent, vortexed for 1 min, and sonicated for 20 min in an ultrasonic bath (Fisher Scientific FS30), following established procedures [26].

### 2.3 Quantification of 10‐HDA in RJ by HPLC

For high‐performance liquid chromatography (HPLC) analysis, 25 mg of fresh RJ or 10 mg of lyophilized RJ was dissolved in 10 mL of methanol:water (50:50, v/v) and sonicated for 30 min. Samples were centrifuged at 1400 × g for 10 min, filtered through 0.45 µm syringe filters, and transferred to autosampler vials. Recovery of 10‐HDA was not determined; however, three syringe filter membrane materials (SFCA, Nylon, PES) were tested and showed no difference in measured 10‐HDA (data not shown). A 10‐HDA stock solution (400 µg/mL) was prepared and serially diluted to generate calibration standards ranging from 1.56 to 200 µg/mL. The standard curve was linear throughout this concentration range (*r* ^2^ = 0.9996). Since all experimental samples showed 10‐HDA concentrations between 40 and 90 µg/mL, which were well above the lowest calibration point, limits of detection and of quantification were not determined.

HPLC analyses were performed using an Agilent 1260 Infinity II system equipped with a diode array detector and a Poroshell 120 EC‐C18 column (4.6 × 100 mm, 2.7 µm). The detection wavelength was set at 235 nm, with an injection volume of 10 µL and a flow rate of 1.0 mL/min. The mobile phase consisted of solvent A (54% deionized water, 23% methanol, 23% acetonitrile, 0.0025% H_3_PO_4_) and solvent B (5% deionized water, 32% acetonitrile, 63% methanol, 0.01% H_3_PO_4_). The gradient program was as follows: 0% B for 3 min, linear increase to 100% B over 1 min, hold at 100% B for 3 min, return to 0% B over 1 min, followed by re‐equilibration at 0% B for 5 min. Under these conditions, 10‐HDA eluted at approximately 2.9 min with compound identification assured by comparing retention time and UV spectrum against a pure 10‐HDA standard, and 10‐HDA was baseline resolved in all samples.

### 2.4 Total Phenolic Content (TPC) Determination

TPC was determined using the Folin–Ciocalteu method with slight modifications, as previously described [27]. Briefly, 100 µL of methanolic RJ extract (50 mg/mL) was mixed with 500 µL of deionized water and 100 µL of Folin–Ciocalteu reagent. After 6 min of incubation at room temperature, 1 mL of 7% (w/v) sodium carbonate solution and 500 µL of deionized water were added. The reaction mixtures were incubated in the dark for 90 min, after which 100‐µL aliquots were transferred in triplicate to 96‐well microplates. Absorbance was measured at 760 nm using a microplate reader. TPC values were expressed as milligrams of gallic acid equivalents per gram of RJ (mg GAE/g) based on a gallic acid calibration curve (*y* = 1.1193 *x* + 0.0429, *R* ^2^ = 0.998).

### 2.5 Total Flavonoid Content (TFC) Determination

TFC was assessed using the aluminum chloride colorimetric method, as previously reported [27]. Briefly, 100 µL of methanolic RJ extract (50 mg/mL) was mixed with 500 µL of deionized water and 100 µL of 5% (w/v) sodium nitrate solution. After 6 min, 150 µL of 10% (w/v) aluminum chloride solution was added. Following a further 5 min incubation, 200 µL of 1 M sodium hydroxide was added to the mixture. Subsequently, 100 µL of each reaction mixture was transferred in triplicate to 96‐well microplates, and absorbance was measured at 510 nm. TFC values were expressed as milligrams of quercetin equivalents (QCE) per gram of RJ (mg QCE/g) using a quercetin calibration curve (*y* = 0.1179 x + 0.2234, *R* ^2^ = 0.9846).

### 2.6 Antiradical Activity (DPPH Assay)

Free radical scavenging activity was evaluated using the DPPH assay, as described by Sambou et al. [28]. Briefly, 200 µL of DPPH solution (250 µM in methanol) was mixed with RJ extracts to achieve a final RJ concentration of 5 mg/mL. The mixtures were shaken and incubated in the dark at room temperature for 30 min. Absorbance was measured at 517 nm. Ethanol was used as the blank, and ascorbic acid served as the positive control.

### 2.7 Antioxidant Activity Assay

Antioxidant activity was assessed by measuring the inhibition of linoleic acid oxidation, as previously described by Boudreau et al. [29]. Fresh and lyophilized RJ samples (final concentration of 1 mg/mL) were added to a reaction mixture containing phosphate‐buffered saline (5 mM, pH 7.4) supplemented with 0.05% Tween 20 and 0.16 mM linoleic acid (Cayman Chemical). Lipid oxidation was initiated by the addition of 2 mM 2,2′‐azobis(2‐amidinopropane) dihydrochloride (AAPH). Aliquots (100 µL) of the reaction mixtures were transferred to UV‐transparent, flat‐bottom 96‐well plates. Control conditions included reactions without linoleic acid (matrix subtraction) and reactions without AAPH (no oxidation control). Following AAPH addition, the rate of lipid oxidation was monitored by measuring absorbance at 234 nm every 5 min for 180 min using a Synergy H1 Hybrid microplate reader (Biotek). Antioxidant activity was expressed as the percentage inhibition of linoleic acid oxidation, calculated using the following equation: Inhibition (%) = [1 – (rate of absorbance change with test sample/rate of absorbance change with solvent control)] × 100.

### 2.8 Cell Viability Assay

Jurkat (human acute T‐cell leukemia; ATCC TIB‐152, RRID: CVCL_0065) and Reh (human acute lymphocytic leukemia; ATCC CRL‐8286, RRID: CVCL_1650) cell lines were obtained from the American Type Culture Collection (ATCC; Manassas, VA, USA). Cell viability was evaluated using the CellTiter‐Blue assay (Promega, Madison, WI, USA), as previously described by Riss et al. [30]. Jurkat and Reh cells (1 × 10^4^ cells per well) were seeded in 96‐well plates in 100 µL of RPMI 1640 medium supplemented with 10% fetal bovine serum (FBS) and 1% penicillin–streptomycin. Cells were treated with RJ samples at a final concentration of 200 µg/mL and incubated for 72 h at 37°C. Following incubation, 20 µL of CellTiter‐Blue reagent was added to each well, and plates were incubated for 1 h (Jurkat cells) or 2 h (Reh cells). Fluorescence was measured using a Synergy H1 Hybrid microplate reader.

### 2.9 Apoptotic Cell Death Assay

Jurkat and Reh cells were seeded in 12‐well plates at a density of 1 × 10^6^ cells per well in 2 mL of RPMI 1640 medium supplemented with 10% FBS and 1% penicillin‐streptomycin (100 µg/mL each). RJ samples were added to achieve a final concentration of 200 µg/mL, and cells were incubated for 72 h. Apoptotic cell death was assessed by flow cytometry using Annexin V and Zombie Aqua staining (BioLegend). Briefly, 500 µL of cell suspension was incubated for 15 min at room temperature in the dark with 100 µL of staining master mix containing Annexin V binding buffer (98.5 µL), Annexin V‐647 (1 µL), and Zombie Aqua (0.5 µL). Samples were analyzed using an Attune NxT flow cytometer (Thermo Fisher Scientific) with Attune NxT Software version 4.2.0. Gates for Annexin V‐647 and Zombie Aqua were set using untreated cells and cells treated with 10 µg/mL of propolis extract as a positive control [25]. No compensation was necessary since there was no overlap in emission spectra using the red and violet lasers for these analyses.

### 2.10 5‐LO Product Biosynthesis Assay

The anti‐inflammatory activity of RJ samples was evaluated using a cellular 5‐LO product biosynthesis assay, as previously described by Sambou et al. [28]. Briefly, HEK293 cells stably co‐transfected with expression vectors encoding 5‐LO and 5‐LO‐activating protein (FLAP) [31] were cultured in Dulbecco's Modified Eagle's Medium (DMEM) supplemented with 10% FBS and 1% penicillin‐streptomycin, and maintained at 37°C in a humidified incubator with 5% CO_2_. Cells were harvested by trypsinization and resuspended in Hank's Balanced Salt Solution (HBSS) containing 1.6 mM CaCl_2_ at a density of 1 × 10^6^ cells/mL.

Cell suspensions were preincubated with fresh or lyophilized RJ samples at a final concentration of 400 µg/mL for 5 min at 37°C. Leukotriene biosynthesis was then stimulated by the simultaneous addition of calcium ionophore A23187 (10 µM) and arachidonic acid (10 µM), followed by incubation for 15 min at 37°C. Reactions were terminated by the addition of 0.5 volumes of ice‐cold stop solution (methanol:acetonitrile, 1:1, v/v) containing 200 ng/mL of 19‐hydroxy prostaglandin B2 (19‐OH PGB2) as an internal standard. Samples were vortexed and stored at −80°C to ensure complete protein denaturation. Lipid metabolites were subsequently analyzed by reverse‐phase high‐performance liquid chromatography (RP‐HPLC), as previously described by Robichaud et al. [32].

### 2.11 Statistical Analysis

Statistical analyses were performed using GraphPad Prism version 10.6.1 (GraphPad Software, San Diego, CA, USA). Detailed descriptions of the statistical tests applied are provided in the corresponding figure and table legends. Prior to analyses, normality of data was tested and if a normal distribution was not attained, log‐transformed data were evaluated for normality and if attained, data were then analyzed. Data are presented as means ± SD. One‐way and two‐way ANOVA tests were performed, and if significance was obtained (*p* < 0.05), these were followed by Tukey or Dunnett's multiple comparison analyses as indicated in the figure legends. Two‐way ANOVA were performed on cellular assays using a randomized block approach with the treatment and day of experiment as independent variables. For 10‐HDA analyses, three separate extractions of the pooled RJ samples were performed, and each extraction was considered as a separate experiment “n.” For TPC/TFC, antioxidant and anti‐radical analyses, three separate fractions of the pooled RJ samples were resuspended in methanol and each was considered as separate experiment “n.” For apoptosis, cell viability and 5‐LO inhibition, two to four separate fractions of the pooled RJ samples were resuspended in DMSO on different days, and each was considered as a separate experiment “n.” Since the apoptosis assays were only executed twice, data are presented as averages ± range and statistical analyses were not performed.

## 3 Results and Discussion

### 3.1 Content of the Fatty Acid 10‐HDA

The fatty acid 10‐HDA is widely regarded as the molecular fingerprint of RJ, as it is unique to this bee product and absent from other hive‐derived matrices [9, 33, 34]. From a chemical and biological standpoint, 10‐HDA represents a key low‐molecular‐weight metabolite that integrates information on biosynthetic capacity, genetic background, and environmental conditions. Its concentration is therefore routinely used as an indicator of RJ purity and overall quality, and it has been associated with several biological activities, including antimicrobial, immunomodulatory, and cytotoxic effects [35, 36].

**FIGURE 1:**
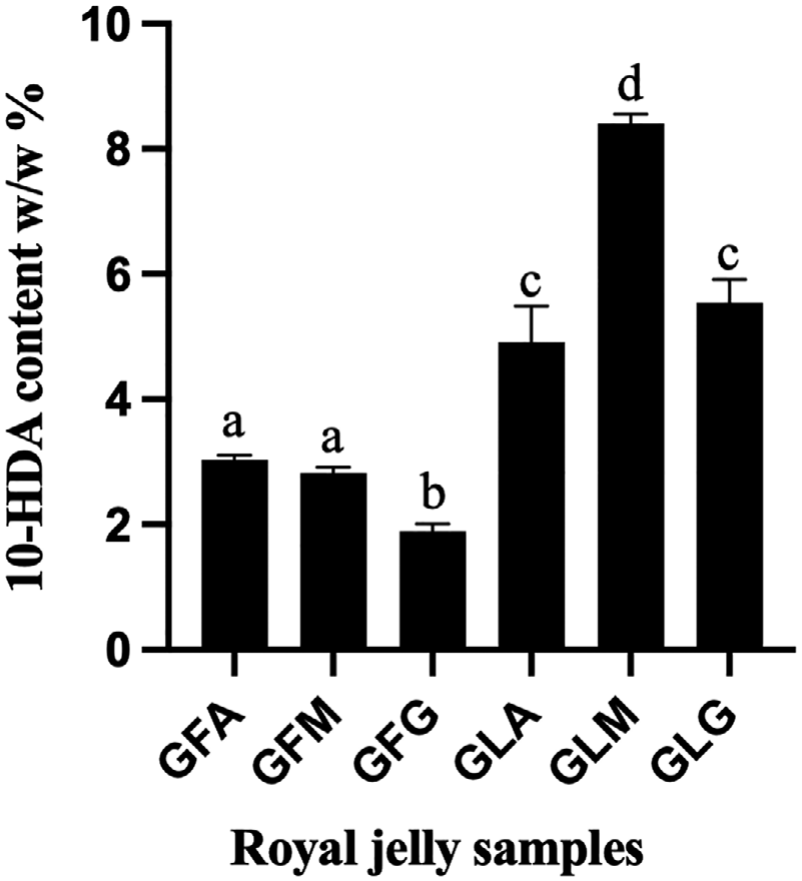
Content of 10‐hydroxy‐2‐decenoic acid (10‐HDA, g/100 g) in fresh and lyophilized Algerian royal jelly (RJ) samples. Data represent means ± SD of three independent experiments. Different superscript letters indicate statistically significant differences (*p* < 0.05) as determined by one‐way ANOVA followed by Tukey's multiple comparisons test.

In the present study, the 10‐HDA content of fresh and lyophilized RJ samples collected from three geographically and ecologically distinct regions of Algeria was quantified by HPLC (Figure 1). Fresh RJ samples exhibited mean 10‐HDA contents ranging from 1.9% to 3.0% (w/w). Samples GFA (Annaba) and GFM (Médéa) displayed the highest concentrations (3.03% and 2.82%, respectively), whereas GFG (Ghardaïa) showed a lower value of 1.9% (w/w). These values fall within the range previously reported for RJ samples from diverse geographic origins, including Australia, Bulgaria, China, Italy, Kenya, and South America (0.8%–3.4% w/w) [34, 37, 38, 39], underscoring the chemical comparability of Algerian RJ to internationally commercialized products.

An important strength of this work lies in the integration of chemical profiling with honeybee biodiversity. Higher 10‐HDA levels have been associated with superior RJ quality and potential health benefits [37], and inter‐sample variability has been attributed, at least in part, to honeybee subspecies differences. The higher 10‐HDA contents observed in GFA and GFM are consistent with their production by *A. m. intermissa*, whereas the lower level measured in GFG may reflect biosynthetic differences associated with *A. m. sahariensis* [5, 6]. This interpretation is further supported by genetic analyses confirming that all populations belong to the native African lineage A while retaining subspecies‐specific characteristics [25]. Together, these findings provide preliminary insights into how intraspecific biodiversity within *A. mellifera* can influence the chemical signature of RJ at the molecular level. However, the respective contributions of subspecies, environmental conditions, and colony‐level factors cannot be clearly disentangled in the present study.

Lyophilized RJ samples displayed substantially higher 10‐HDA contents, ranging from 4.9% to 8.4%, with sample GLM exhibiting the highest concentration. Considering that fresh RJ typically contains 60%–70% water, these results suggest that lyophilization concentrates 10‐HDA on a dry‐weight basis while preserving the overall regional pattern observed among samples. The more modest increase observed in GLA could be related to a lower initial water content in the corresponding fresh RJ sample, although moisture content was not directly measured, and this remains a hypothesis. These observations are consistent with previous reports demonstrating that lyophilization maintains key RJ constituents [24, 36]. In the context of the present study, the inclusion of lyophilized samples mainly served to confirm that the comparative trends observed in fresh Algerian RJ were also detectable after processing.

Despite these strengths, this study has certain limitations. In particular, the limited sampling design and the pooling of samples at the apiary level restrict the ability to disentangle the respective contributions of subspecies, geography, and colony‐level variability. In addition, parameters such as production conditions, environmental factors, and handling practices were not experimentally controlled and may have contributed to the observed variability. While 10‐HDA is a well‐established quality marker, RJ is a chemically complex matrix, and its biological properties likely arise from synergistic interactions between multiple constituents, including proteins and phenolic compounds. Another limitation is the sample preparation method. Methanol was used for characterization analyses, and DMSO‐based preparations were used for cell assays, so the results mainly reflect the RJ fraction that could be extracted or dispersed under these conditions. Future studies combining targeted and untargeted metabolomic approaches, as well as expanded sampling across seasons and additional subspecies, would further clarify the relative contribution of 10‐HDA to RJ bioactivity and better capture the contribution of underrepresented RJ constituents, especially proteins. Nonetheless, the present findings demonstrate that Algerian RJ, produced by native honeybee subspecies and subjected to controlled processing, represents a chemically robust and biodiverse natural product of relevance to both fundamental and applied research.

### 3.2 Total Phenolic and Flavonoid Contents

The estimated TPC of the RJ samples was expressed as gallic acid equivalents (GAE). As shown in Table 1, fresh RJ samples from Annaba (GFA) and Médéa (GFM) exhibited comparable TPC values, whereas the sample from Ghardaïa (GFG) showed a significantly higher phenolic content. A similar distribution pattern was observed in the corresponding lyophilized samples. When placed in a broader geographical context, the TPC values measured for Algerian RJ were comparable to those reported for both local and commercial RJ samples from Romania (14.6–39.9 and 15.4–32.5 mg GAE/g, respectively) and Japan (21.2–22.8 mg GAE/g) [40]. Moreover, these values exceeded those reported for RJ samples from Turkey (0.592 mg GAE/g) [11], highlighting the relatively rich phenolic profile of Algerian RJ.

**TABLE 1:** Total phenolic content (TPC), total flavonoid content (TFC), and antiradical and antioxidant activities of Algerian royal jelly (RJ) samples.

	TPC (mg GAE/g RJ)	TFC (mg QCE/g RJ)	Antiradical activity (5 mg RJ/mL) (% inhibition)	Antioxidant activity (1 mg RJ/mL) (% inhibition)
GFA	16.9 ± 2.6^a^	0.18 ± 0.01^a^	13.4 ± 0.8^a^	28.6 ± 2.9^a,b^
GFM	21.8 ± 1.4^a^	0.34 ± 0.15^a^	15.6 ± 0.9^a,b^	10.9 ± 2.7^a^
GFG	32.4 ± 2.8^b^	0.02 ± 0.01^b^	20.1 ± 0.7^a,b^	19.7 ± 8.8^a,c^
GLA	47.3 ± 1.4^c^	1.85 ± 0.17^c^	21.0 ± 0.5^a,b^	56.0 ± 19.2^d^
GLM	53.7 ± 1.3^c^	3.07 ± 0.74^c^	15.5 ± 0.6^a,b^	25.5 ± 4.0^a,b^
GLG	64.9 ± 4.4^d^	2.78 ± 0.73^c^	28.3 ± 0.5^b^	44.0 ± 8.4^b,c.d^
Ascorbic acid (0.018 mg/mL)	ND	ND	82.9 ± 0.7	ND
Zileuton (10 µM)	ND	ND	ND	81.1

Values are expressed as means ± SD of three independent experiments. Within each column, values *without at least one common superscript letter* are significantly different (*p* < 0.05) as determined by one‐way ANOVA followed by Tukey's multiple comparisons test. Log‐transformed data were used when normality of residuals was not achieved. Abbreviations: GAE, gallic acid equivalents; QCE, quercetin equivalents; ND, not determined.

From a chemical biodiversity perspective, these findings underscore the influence of ecological and geographical factors on the accumulation of phenolic compounds in RJ. Although RJ is not traditionally regarded as a phenolic‐rich bee product, its estimated phenolic fraction may contribute to antioxidant capacity and interact synergistically with other constituents such as fatty acids and proteins. The elevated TPC observed in the GFG samples, produced in an arid desert environment by *A. m. sahariensis*, suggests that environmental stressors or subspecies‐specific metabolic pathways may modulate the transfer or synthesis of phenolic compounds in RJ.

The estimated TFC, expressed as QCE, revealed low but quantifiable flavonoid levels in fresh RJ samples, ranging from 0.02 ± 0.01 to 0.34 ± 0.15 mg QCE/g, with no statistically significant differences among samples (Table 1). These values are consistent with those reported by El‐Guendouz et al. [27], who documented flavonoid contents between 0.1 and 0.5 mg QCE/g in RJ. The relatively low estimated flavonoid content in RJ has been attributed to its biosynthetic origin: RJ is secreted by young worker bees that consume pollen and honey, which are the primary dietary sources of plant‐derived polyphenols [41].

Despite their low estimated concentrations, the variability observed in TPC and TFC among samples supports the concept that RJ composition is shaped by multiple interacting factors, including honey bee subspecies, geographical origin, climatic conditions, harvesting period, storage, and processing methods [9, 10, 41, 42]. In this context, the present study design does not allow for the isolation of individual variables, and the relative contribution of each factor remains to be clarified in future studies involving larger and more controlled sampling. Notably, in the present study, RJ samples from Annaba and Médéa originated from *A. m. intermissa*, whereas RJ from Ghardaïa was produced by *A. m. sahariensis*, providing a natural framework to explore potential relationships between subspecies, environmental conditions, and RJ chemistry. Of note, the regional trends observed for RJ phenolic and flavonoid contents did not mirror those previously reported for propolis collected from the same hives, where Médéa and Annaba propolis exhibited the highest phenolic and flavonoid levels, respectively [25]. This divergence highlights the product‐specific nature of chemical biodiversity within the hive and cautions against extrapolating compositional patterns across different bee products [3, 4].

TPC and TFC trends were broadly conserved between fresh and lyophilized samples, indicating that sample form did not obscure the comparative differences observed among Algerian RJ samples. This observation is in agreement with earlier findings on freeze‐dried RJ [36]. Nevertheless, the present work is limited to global phenolic and flavonoid measurements. Future studies employing targeted and untargeted metabolomic approaches would enable the identification of individual phenolic compounds and their potential interactions with other RJ constituents, thereby providing a more comprehensive understanding of how chemical diversity translates into biological activity.

### 3.3 Antiradical and Antioxidant Activity

The antiradical and antioxidant properties of the RJ samples were evaluated using two complementary assays: DPPH free radical scavenging and inhibition of linoleic acid oxidation. Together, these assays provide insight into both direct radical‐quenching capacity and the ability to limit lipid peroxidation in a biologically relevant system. As shown in Table 1, all RJ samples exhibited comparable DPPH radical scavenging activity, with the exception of the lyophilized Ghardaïa sample (GLG), which demonstrated higher activity than the other samples. Despite this relative difference, the overall antiradical activities of all RJ samples were weak when compared to the positive control, ascorbic acid.

The DPPH scavenging activities observed in this study are consistent with previously reported values for RJ from different geographical origins. Mokaya et al. [37] and Kolayli et al. [43] reported IC_50_ values ranging from 102 to 354 mg/mL for African and Turkish RJ samples. While our study measured DPPH activity at a single concentration (5 mg/mL) and did not generate full concentration‐response curves or IC_50_ values, the observed relative activities suggest that the antiradical capacity of Algerian RJ is modest, in line with previous reports. This consistency across studies reinforces the reliability of the analytical approach and suggests that modest antiradical capacity is an intrinsic characteristic of RJ rather than a limitation of sample origin or processing.

Antioxidant activity assessed by inhibition of linoleic acid oxidation (Table 1) tended to be higher in lyophilized RJ samples than in their fresh counterparts, although the overall antioxidant effects remained limited, even at the relatively high RJ concentrations tested. The antioxidant potential of RJ has been attributed to a complex interplay of constituents, including 10‐HDA [38, 44], phenolic and flavonoid compounds [37, 38, 45], proteins and peptides [10, 15, 46], as well as vitamins and trace elements [47, 48, 49]. The relatively low antioxidant efficacy observed here likely reflects the comparatively low abundance of phytochemicals in RJ, as previously suggested by Mokaya et al. [37] and highlights the importance of considering both concentration and chemical diversity when evaluating antioxidant performance. Furthermore, variations in production conditions, sampling handling, and environmental factors may also have influenced the measured activity.

A notable strength of this study is the direct comparison between fresh and lyophilized RJ samples derived from distinct ecological regions. The broadly similar antiradical and antioxidant trends observed across sample forms suggest that processing did not substantially alter the overall comparative profile among regions. In this respect, fresh and lyophilized RJ can be viewed as complementary matrices that mutually support the robustness of the observed bioactivity patterns [36].

At the same time, these results emphasize an important limitation: RJ is not a potent antioxidant when compared to other bee products or natural matrices. For example, propolis exhibits IC_50_ values in the low microgram per milliliter range (3–5 µg/mL), making it approximately three orders of magnitude more potent than RJ in comparable assays [25]. This contrast underscores the product‐specific nature of chemical functionality within the hive and cautions against generalizing antioxidant claims across different bee‐derived substances.

Based on our findings, we suggest that future research should move beyond global antioxidant assays to explore structure–activity relationships among individual RJ constituents and their potential synergistic interactions. Advanced metabolomic profiling, coupled with bioactivity‐guided fractionation, could help identify minor compounds that contribute disproportionately to antioxidant effects. Such approaches would further clarify the role of chemical diversity in shaping the biological properties of RJ and strengthen its positioning within the broader landscape of natural products research.

### 3.4 Cell Viability, Apoptosis, and Cell Death Assays

**FIGURE 2:**
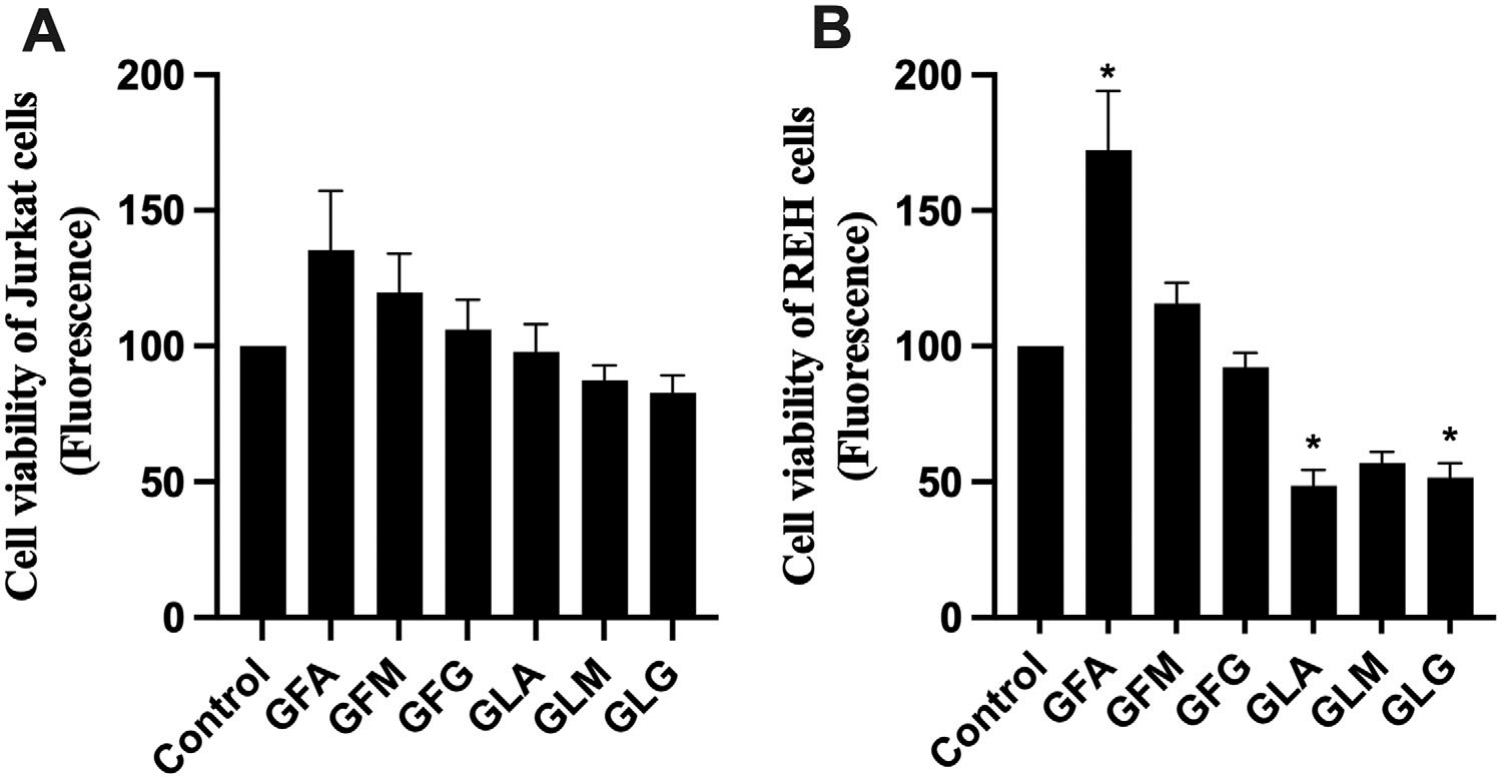
Effects of RJ on cell viability in Jurkat (A) and Reh (B) cells after 72 h treatment (200 µg/mL), measured by the CellTiter‐Blue assay. Data represent means ± SD of three independent experiments, each performed in duplicate. * Significantly different from Control (DMSO) at *p* < 0.05, as determined by two‐way ANOVA with Dunnett's multiple comparisons test on non‐normalized data.

The antiproliferative and pro‐apoptotic effects of Algerian RJ samples were evaluated using two human acute lymphoblastic leukemia cell lines, Jurkat (T‐cell) and Reh (B‐cell). This dual‐cell‐line approach represents a strength of the present study, as it allows the exploration of cell‐type‐specific responses to a chemically complex natural product. As shown in Figure 2, none of the RJ samples significantly affected the viability of Jurkat cells under the experimental conditions employed. In contrast, lyophilized RJ samples induced a significant reduction in Reh cell viability. Interestingly, fresh RJ from the Annaba region increased the viability signal in Reh cells under the assay conditions, which may reflect increased metabolic activity rather than a true proliferative or survival‐promoting effect and would require confirmation by an orthogonal method. This response was abolished following lyophilization, indicating that processing may alter the balance between constituents influencing the assay readout or growth‐promoting and growth‐inhibitory constituents. The molecular basis of this differential cellular response remains unclear and warrants further investigation.

To better understand the mechanisms underlying these viability changes, apoptotic cell death was assessed by flow cytometry using Annexin V and Zombie Aqua staining [50]. As shown in Table 2, treatment with RJ preparations at a final concentration of 200 µg/mL did not appear to induce an increase in apoptotic cell populations in either Jurkat or Reh cells relative to untreated controls (DMSO). Instead, the observed reduction in the proportion of viable (Annexin V^−^/Zombie Aqua^−^) cells was accompanied by an increase in necrotic or membrane‐compromised cells (Annexin V^−^/Zombie Aqua^+^). These findings suggest that the decrease in cell viability observed in Reh cells is more likely attributable to nonspecific cytotoxicity rather than the activation of programmed apoptotic pathways. It should be noted that all cell‐based assays were performed at a single concentration (200 µg/mL), providing preliminary screening data rather than full concentration–response information. This approach is consistent with established practices for initial anticancer screening and can be clinically relevant; for example, the U.S. National Cancer Institute (NCI) NCI‐60 Cell One‐Dose screening for potential therapeutic molecules is conducted at a single concentration of 10 µM (https://dtp.cancer.gov/discovery_development/nci‐60/methodology.htm), and crude plant extracts showing IC_50_ values at concentrations of 20 µg/mL or lower are typically considered active [51, 52]. The modest and cell‐type‐dependent antiproliferative effects observed here are consistent with previous reports describing limited or statistically non‐significant growth inhibition by RJ in astrocytoma, glioblastoma multiforme, astroglia, and colorectal cancer cell models [53, 54]. In contrast, stronger antiproliferative effects have been reported in other cancer cell lines [44, 55, 56, 57], often in a dose‐dependent manner [58]. These comparisons highlight that cellular context, concentration, and exposure time are key factors influencing RJ bioactivity, while our findings emphasize that Algerian RJ exhibits modest activity under the tested conditions.

**TABLE 2:** Average percentages of Jurkat and Reh cells in each quadrant following Annexin V and Zombie Aqua staining after 72 h treatment with RJ samples (200 µg/mL).

	Control (DMSO)	GFA	GFM	GFG	GLA	GLM	GLG
Jurkat cells							
A− Z−	76.4 (71.3–81.5)	76.4 (72.3–80.4)	77.5 (71.1–83.8)	75.3 (69.2‐81.3)	69.9 (68.9–70.8)	72.0 (69.8–71.1)	69.9 (68.9–70.8)
A+ Z−	10.7 (6.0–5.3)	6.2 (5.9–6.5)	8.1 (6.1–9.9)	8.0 (7.7‐8.2)	7.9 (7.5–8.1)	9.0 (8.2–9.7)	6.7 (5.9–7.4)
A+ Z+	11.5 (10.8–12.0)	13.3 (11.6–14.9)	10.5 (5.5–15.3)	11.0 (8.9‐13.0)	12.3 (10.4–14.0)	10.6 (7.6–13.5)	12.7 (11.5–13.8)
A− Z+	1.4 (1.2–1.5)	4.0 (1.9–6.1)	4.0 (0.6–7.2)	5.7 (2.0–9.4)	10.0 (8.7–11.1)	8.4 (6.7–10.0)	10.7 (7.9–13.5)
REH cells							
A− Z−	94.4 (93.5–95.3)	90.7 (88.5–92.9)	91.0 (89.1–92.7)	87.2 (86.9–87.5)	85.5 (85.3–85.6)	88.8 (87.8–89.7)	90.1 (88.6–91.5)
A+ Z−	1.9 (1.8–1.9)	1.8 (1.6–2.0)	2.0 (1.8–2.0)	2.6 (1.4–3.6)	2.2 (1.9–2.4)	2.8 (2.2–3.3)	2.7 (2.1–3.1)
A+ Z+	3.6 (2.8–4.4)	4.3 (3.3–5.2)	3.8 (2.5–5.1)	3.6 (3.3–3.8)	4.0 (3.3–4.6)	3.8 (3.4–4.2)	3.6 (2.7–4.5)
A‐ Z+	0.07 (0.05–0.08)	3.1 (1.9–4.2)	3.3 (2.8–3.7)	6.6 (5.5–7.7)	8.3 (7.2–9.3)	4.5 (4.5–4.6)	3.6 (3.5–3.6)

*Note*: Cells were cultured in RPMI‐1640 medium with 10% FBS. Post‐treatment, cells were stained with Annexin V‐647 and Zombie Aqua and analyzed by flow cytometry. Values represent the mean and range (min‐max) from two independent experiments, each performed in duplicate. Abbreviations: A = Annexin V; Z = Zombie Aqua.

The fatty acid 10‐HDA has frequently been proposed as a key mediator of RJ's anticancer activity [10, 21, 26]. In the current study, Algerian RJ showed limited antiproliferative activity despite the presence of 10‐HDA, suggesting that other constituents or interactions contribute [59].

Taken together, the present findings indicate that Algerian RJ exhibits limited antiproliferative activity under the tested conditions and does not robustly induce apoptosis in leukemia cell lines. These results reinforce the consensus that RJ is unlikely to function as an effective anticancer monotherapy and should not be considered a stand‐alone therapeutic agent [53, 54]. Nevertheless, from a chemical biodiversity perspective, the differential responses observed between cell lines and processing states point to subtle, composition‐dependent biological effects. Future studies should focus on dose‐response relationships, fractionation‐guided bioassays, and molecular pathway analyses to clarify the mechanisms underlying RJ–cell interactions and to define more precisely the boundaries of its biological activity.

### 3.5 5‐LO Product Biosynthesis Assay in HEK293 Cells

Inflammation is a fundamental component of the innate immune response; however, its dysregulation can lead to tissue damage and the progression of chronic inflammatory diseases [60]. A central mediator of inflammatory signaling is the 5‐LO pathway, which catalyzes the conversion of arachidonic acid into leukotrienes that exert potent pro‐inflammatory effects. Owing to its pivotal role, 5‐LO is a well‐validated molecular target for anti‐inflammatory drug discovery. Despite extensive research efforts, only one direct 5‐LO inhibitor, zileuton, has been approved for clinical use in the United States, and its broader application remains limited due to unfavorable pharmacokinetics and hepatotoxicity [61]. These limitations have fueled continued interest in identifying alternative 5‐LO modulators, including those derived from natural products.

**FIGURE 3:**
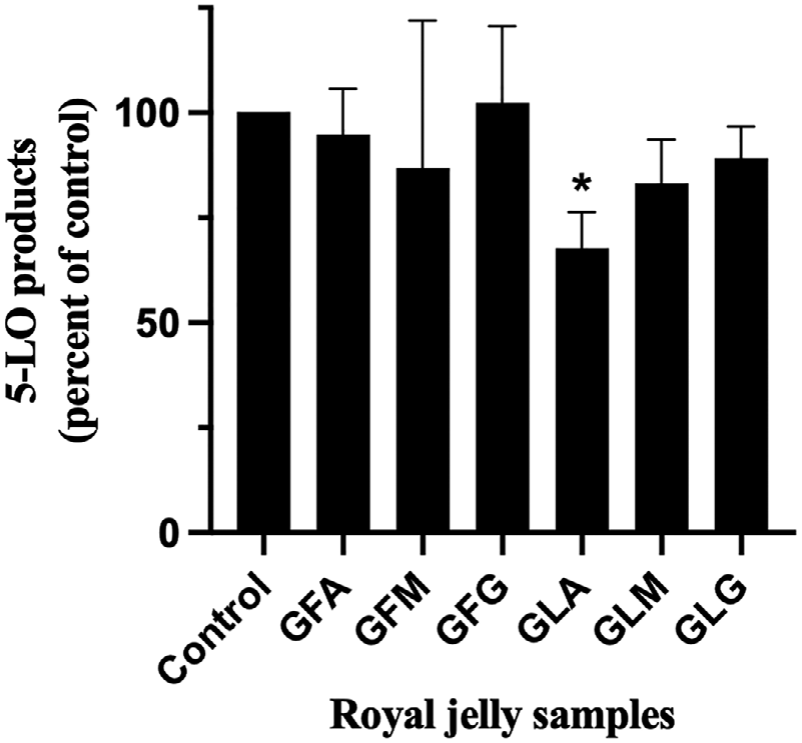
Inhibition of 5‐lipoxygenase (5‐LO) product biosynthesis in HEK293 cells treated with fresh and lyophilized RJ samples (400 µg/mL). Data are expressed as percentage of 5‐LO metabolite production relative to the DMSO solvent control. Values represent means ± SD of four independent experiments, each performed in duplicate. Statistical significance (*p* < 0.05) was determined by two‐way ANOVA on non‐normalized data.

In the present study, the ability of fresh and lyophilized RJ samples to inhibit 5‐LO product biosynthesis was evaluated using HEK293 cells stably expressing human 5‐LO and 5‐LO‐activating protein (FLAP) [31]. This whole‐cell assay represents a significant methodological strength, as it captures the integrated biochemical and cellular context of leukotriene biosynthesis and provides a more physiologically relevant assessment of inhibitory potential than enzyme‐based assays employing non‐human lipoxygenase sources. The 5‐LO inhibition assay was conducted at a single concentration (400 µg/mL), serving as an initial screening to identify potential activity. As shown in Figure 3, among all tested samples, only lyophilized RJ from the Annaba region exhibited detectable inhibition of 5‐LO product biosynthesis, while all other RJ preparations were inactive at the concentration tested (400 µg/mL). By comparison, the 5‐LO inhibitor zileuton inhibits the biosynthesis of 5‐LO products with an IC_50_ of ∼2 µM (∼0.5 µg/mL) in this cell‐based assay [62]. These results indicate that under the tested conditions, RJ is largely inactive toward 5‐LO.

These findings contrast with previous reports describing anti‐inflammatory effects of RJ, which have largely focused on the suppression of pro‐inflammatory cytokine production in macrophage models, often in a dose‐dependent manner and without apparent cytotoxicity [44]. Additionally, El‐Guendouz et al. [27] demonstrated that RJ samples from Morocco, Spain, and Portugal could inhibit lipoxygenase activity derived from soybean extracts, albeit with relatively high IC_50_ values in the low mg/mL range. The discrepancy between these studies and the present results likely reflects differences in experimental systems, including enzyme source, assay format, and biological complexity. Notably, the use of human 5‐LO in a cellular context in the current study provides a more stringent and therapeutically relevant test of anti‐inflammatory potential.

Given the minimal inhibition observed at concentrations up to 400 µg/mL, the present data indicate that RJ is not an effective inhibitor of 5‐LO product biosynthesis. The minimal effect of RJ at 400 µg/mL, relative to the sub‐micromolar activity of zileuton in the same cellular assay [62], confirms that the observed inactivity of RJ reflects low inhibitory potency rather than a non‐responsive assay. This comparison strengthens the conclusion that RJ is unlikely to directly inhibit the 5‐LO pathway and suggests that any anti‐inflammatory effects may be mediated through alternative mechanisms. This conclusion is further reinforced by direct comparison with propolis extracts obtained from the same geographical regions, which inhibited 5‐LO biosynthesis using the same cellular model as the current study with IC_50_ values in the low microgram per milliliter range (0.6–3.3 µg/mL) [25]. This stark contrast highlights the product‐specific nature of anti‐inflammatory activity among bee‐derived substances and underscores the importance of rigorous, pathway‐specific evaluation.

Together, our results support the view that while RJ is a chemically complex mixture containing bioactive constituents such as 10‐HDA, phenolics, and flavonoids, its anti‐inflammatory effects are unlikely to be mediated through direct inhibition of the 5‐LO pathway. Instead, RJ may exert more subtle immunomodulatory effects via alternative signaling mechanisms. Importantly, because the cell‐based experiments used DMSO‐based RJ preparations, the biological results mainly reflect the RJ fraction extracted under these conditions and may not fully represent the contribution of all native RJ components, especially MRJPs. Furthermore, the limited effects of both fresh and lyophilized Algerian RJ on leukemia cell viability reinforce the conclusion that RJ is unlikely to serve as an effective monotherapy for inflammatory or proliferative diseases. The generally consistent patterns observed between fresh and lyophilized samples further indicate that the main conclusions of this study are robust across sample form, supporting the use of lyophilized RJ as a complementary format for comparative analyses. Future studies should take into account additional factors, such as variations in production conditions, sample handling, and environmental factors, which may also influence the biological activity of RJ, and should focus on elucidating non‐5‐LO‐dependent anti‐inflammatory mechanisms and on defining how specific RJ constituents contribute to immunomodulation within more complex biological systems.

## Author Contributions

**Ahmed Sabri Ayad**: Participated in research design, conducted experiments, performed data analysis, wrote or contributed to the writing and editing of the manuscript. **Mathieu P. A. Hébert**: conducted experiments, performed data analysis. **Jérémie A. Doiron**: conducted experiments, performed data analysis. **Wahida Loucif‐Ayad**: participated in research design, wrote or contributed to the writing and editing of the manuscript. **Tarek Daas**: participated in research design, wrote or contributed to the writing and editing of the manuscript. **Guy Smagghe**: participated in research design, wrote or contributed to the writing and editing of the manuscript. **Mohamed Touaibia**: participated in research design, wrote or contributed to the writing and editing of the manuscript. **Marc E. Surette**: participated in research design, performed data analysis, wrote or contributed to the writing and editing of the manuscript.

## Funding

This research was supported by the Ministry of Higher Education and Scientific Research of Algeria (PRFU project D01N01UN230120220003 to Prof. **W. Loucif‐Ayad**), the New Brunswick Innovation Foundation to Marc E. Surette (RPI_2024_020, and the Natural Sciences and Engineering Research Council of Canada (RGPIN‐2022‐03950) to Mohamed Touaibia.

## Conflicts of Interest

The authors declare no conflicts of interest.

## Data Availability

The data that support the findings of this study are available from the corresponding author upon reasonable request.
